# Hyperinsulinemic hypoglycemia in adolescents: case report and systematic review

**DOI:** 10.1186/s40842-022-00138-x

**Published:** 2022-03-15

**Authors:** M. G. Castillo-López, M. F. Fernandez, N. Sforza, N. C. Barbás, F. Pattin, G. Mendez, F. Ogresta, I. Gondolesi, P. Barros Schelotto, C. Musso, G. E. Gondolesi

**Affiliations:** 1 Diabetes Metabolic department, Hospital Universitario Fundación Favaloro, Buenos Aires, Argentina; 2 General Surgery, Liver, Intestinal and Pancreas Transplant Services, Hospital Universitario Fundación Favaloro, Buenos Aires, Argentina; 3 Anatomo pathology department, Hospital Universitario Fundación Favaloro, Buenos Aires, Argentina; 4 Oncology department Hospital Universitario Fundación Favaloro, Buenos Aires, Argentina; 5 Imaxe Radiology Institute, Buenos Aires, Argentina; 6 Medical Student, Facultad de Medicina, Universidad Favaloro, Buenos Aires, Argentina; 7 General Surgery Department, and Liver, Pancreas and Intestinal Transplant Units., University Hospital, Favaloro Foundation, Avenida Belgrano 1782, 7mo piso (1093), Buenos Aires, Argentina

**Keywords:** Hyperinsulinemic hypoglycemia (HH), Whipple triad, Nesidioblastosis

## Abstract

**Background:**

Hyperinsulinemic hypoglycemia is the most common cause of severe and persistent hypoglycemia in neonates and children. It is a heterogeneous condition with dysregulated insulin secretion, which persists in the presence of low blood glucose levels.

**Case presentation:**

We report a case of a 15 year-old male with hyperinsulinemic hypoglycemia, who underwent a subtotal pancreatectomy after inadequate response to medical therapy. Pathological examination was positive for nesidioblastosis (diffuse β-cell hyperplasia by H-E and immunohistochemical techniques). The patient’s blood glucose levels normalized after surgery and he remains asymptomatic after 1 year of follow-up. The systematic review allowed us to identify 41 adolescents from a total of 205 cases reported in 22 manuscripts, from a total of 454 found in the original search done in PubMed and Lilacs.

**Conclusions:**

Although very well reported in children, hyperinsulinemic hypoglycemia can occur in adolescents or young adults, as it happens in our reported case. These patients can be seen, treated and reported by pediatricians or adult teams either way due to the wide age range used to define adolescence. Most of them do not respond to medical treatment, and subtotal distal pancreatectomy has become the elected procedure with excellent long-term response in the vast majority.

## Introduction

Glucose is a key metabolic substrate for cellular energy metabolism, and insulin is the primary hormone that lowers plasma glucose concentration. Under normal physiological conditions, secretion of insulin from the β cell is precisely regulated to prevent hypoglycemia or hyperglycemia [1]. In neonates and children, hyperinsulinemic hypoglycemia (HH) remains as the most common cause of severe and persistent hypoglycemia [1]. This condition represents a group of clinically, genetically and histologically heterogeneous disorders, characterized by inappropriate insulin secretion from the pancreatic β-cells in the presence of low blood glucose levels. Recent advances in genetics have linked congenital mutations in 9 different genes that play a key role in regulating insulin secretion [2].

Three histopathological patterns of congenital HH have been distinguished:

1. Diffuse Form: is typically characterized by hyperchromatic β-cell enlargement and Hyperplasia [1].
2. Focal Form: is nodular hyperplasia with acinar-ductular complexes confined to a single region of the pancreas, with normal surrounding pancreas [1]. Insulinomas should be considered in the differential diagnosis, especially in older children, adolescents, or adults [1–3].
3. Atypical Form: has been described related to morphological mosaicism. In this histological type some of the islets are hyperplastic with cytoplasm-rich β-cells with enlarged nuclei, and others are shrunken with cells showing little cytoplasm and small nucleus [4].

Diffuse HH occurs in about 40–50% of patients and is due to biallelic recessive or dominant mutations in the genes *ABCC8*, *KCNJ11*, *GLUD1*, *GCK*, *HADH*, *SLC16A1*, *HNF4A*, *HNF1A*, and *UCP2* [1]. The focal form is found in approximately 50% of patients with HH, having a unique cause that involves two independent events: a) the inheritance of a paternal mutation in ABCC8 or KCNJ11 (located in the 11p15.1 region), and b) the somatic loss of the maternal 11p15 allele including the ABCC8 or KCNJ11 region within the focal lesion. It is important to differentiate between diffuse and focal disease before surgery [5].

In newborn babies and young infants, the clinical presentation may be with non-specific hypoglycemic symptoms such as poor feeding, lethargy, jitteriness, and irritability. In adults and adolescence, the HH usually presents itself as hypoglycemia during fasting, exercise, or during the postprandial period [1]. The clinical symptoms are not specific, and they can be easily mistaken for those of an insulinoma [6] since both of them usually present with Whipple’s triad:

- Signs and symptoms of Hypoglycemia
- Resolution of symptoms once glucose level rises
- Low plasma glucose level (< 55 mg/dl).

The diagnosis of HH due to nesidioblastosis is based on the clinical presentation of hypoglycemia, and biochemical profile that arises from the anabolic effects of increased serum insulin concentration. However, the C-peptide concentration is almost always elevated at the time of hypoglycemia and this surrogate marker could be a better indication of dysregulated insulin secretion than serum insulin itself.

Among imaging examinations, ultrasound (US), abdominal computer tomography (CT), positron emission tomography scan (PET scan) and pancreatic and portal venous sampling with calcium stimulation (when available) can be useful to locate a particular lesion, as a differential diagnosis with insulinoma or another insulin-producing tumor in the adult population. However, these imaging tests, such as ultrasound and computed tomography, are to identify the presence of an insulinoma and are not appropriate for use in newborns with hyperinsulinism. A final diagnosis is provided histologically, including the following criteria:

1. Increase in the size and number of β cells within islets of Langerhans
2. Increase in the number of periductal islets
3. Hyperchromatic nuclei abundant clear cytoplasm
4. Microscopic and immunohistochemical exclusion of an insulinoma  [7].
5. Genetic testing.

Although nesidioblastosis continues to be used in adults’ literature, the use of the term in pediatrics has been abandoned, and persistent hyperinsulinemic hypoglycemia (HH) has been reported to be linked to hyperactive pancreatic beta-cells instead of their increased proliferation, however one of the causes of HH is nesidioblastosis.

## Material and methods

We aim, as a primary end-point, to report a clinical case of an adolescent operated on for presenting a HH syndrome together with diffuse hyperfunctioning pancreatic tissue in the PET-TC scan, defined as nesidioblastosis in the analysis done in the pathology of the surgical specimen. He was studied with routine blood tests including determination of chromogranin, glycose, insulin, c-peptide, HbA1c and oral glucose tolerance test (OGTT). Anti-insulin antibody, antithyroid and antinuclear antibodies tests were performed to rule out autoimmune hypoglycemia. Among the imaging studies, fluoro-18-L-dihydroxyphenylalanine (F-DOPA) PET-CT scan was performed.

As a *second end-point*, we performed a systematic review to cover the adolescent-onset of nesidioblastosis, by using PubMed and Lilacs, in order to assess the existing cases, the proposed treatments, as well as follow up and outcomes. We defined the groups as following: Children between 10 and 14 years old, Adolescents between 15 and 21 and Adults when patients were older than 22 years old. Papers containing patients belonging to more than one age group, were categorized according to their youngest patient’s age.

A mesh search was done with the following terms: “Nesidioblastosis”, “adults”, “adolescents” and “children”. Papers were blindly reviewed by 3 researchers and matched to avoid duplications. Then reviewers excluded the ones in which adolescents were not identified within the series, those with incomplete available data, those when primary language was neither English nor Spanish, those with unavailability of manuscripts and finally those papers that despite appearing in the search, had a main topic or purpose different to our pre-established objective.

## Case report

A 15-year-old male (BMI 24.2 Kg/m2) had the first onset of the disease on March 2018, with mild headache and dizziness. During a routine blood test, severe hypoglycemia (26.8 mg/dl) was detected, referred by the patient’s mother. He had no family history of diabetes or any other hereditary disease.

He was studied by a primary physician in his city who indicated an OGTT that could not be completed due to an acute onset of nausea, headache and lightheadedness. The patient underwent metabolic testing (ruling out adrenal insufficiency and hypothyroidism) with the subsequent diagnosis of hyperinsulinemic hypoglycemia. Chromogranin blood levels were 41 ng/ml (Normal reference value: < 100 ng/dl). In our setting it was not possible to correlate studies determining the use of sulfonylureas in urine and blood because the patient was referred from another medical center for surgical conduct, with studies already been carried out. But at referral, spontaneous fasting insulin, C-peptide and HbA1C levels (Table 1), did not support the possibility of having a possible factitious form.

**Table 1:** Pre and postoperative laboratory results: HbA1C, Glycated hemoglobin

Variable	Pre-surgery	Early post-surgery (< 30 days)	Late post-surgery (> 30 days)
**Insulin (**2-15 **uU/ml)**	26.8	8.4	4.3
**C-peptide (**0. 29-2.35 **ng/ml)**	1.11	1.08	0.4
**HbA1C (**4. 8-5.9**%)**	4.9	5.5	5.0
**Glycemia (74-99 mg/dl)**	45^a^	110	91

^a^Measured by finger prick test

**Fig. 1:**
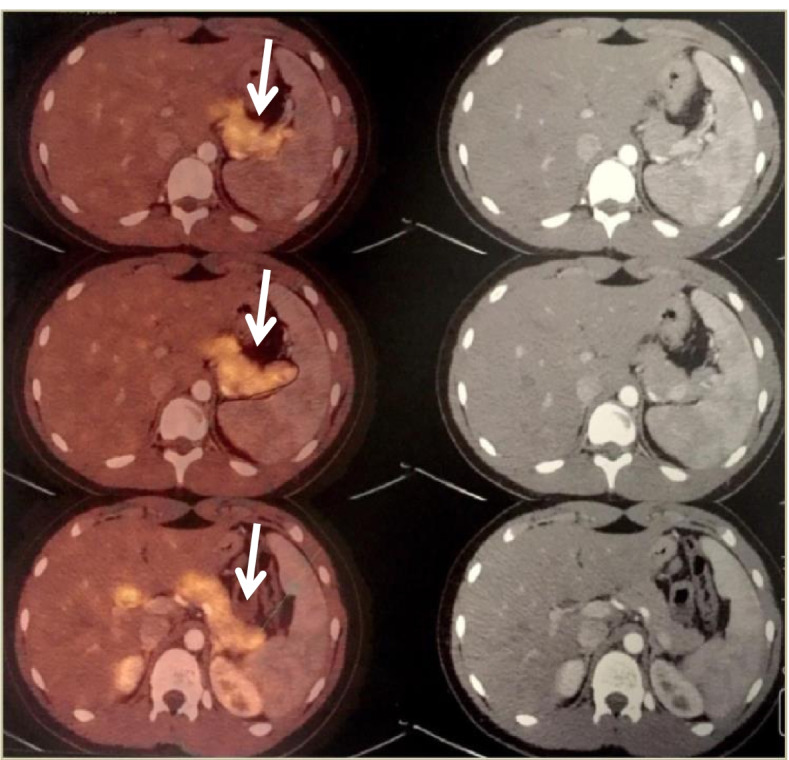
PET-Scan: white arrows show the increased radiotracer uptake distributed all along the pancreatic tissue

Afterwards, a 18-F-DOPA PET-CT scan was performed (Fig. 1), detecting diffuse hyperfunctioning of pancreatic tissue in the body (SUV max. of 4.8) and tail (SUV max of 5.8). A psychological evaluation was indicated for him and his family in order to rule out any factitious behavior or Munchausen syndrome.

Based on the clinical presentation, laboratory studies and PET scan result, the patient was given diazoxide 300 mg per day for 2 months. In spite of the dietary and pharmacological treatment, he continued having clinical manifestations of hypoglycemia at least 4 nights a week with finger stick values of glucose of < 40 mg/dl. For this reason, he was referred to our institution, where a multidisciplinary team evaluated him. After a comprehensive assessment, it was decided that the best approach was to perform a subtotal pancreatectomy (80-90%), as the international literature recommends for patients with HH that do not respond to the medical treatment [8–10].

An exploratory laparoscopy and intraoperative ultrasound were carried out without identifying any macroscopic lesion. Therefore, it was decided to perform an 80% distal pancreatectomy with spleen preservation.

**Fig. 2:**
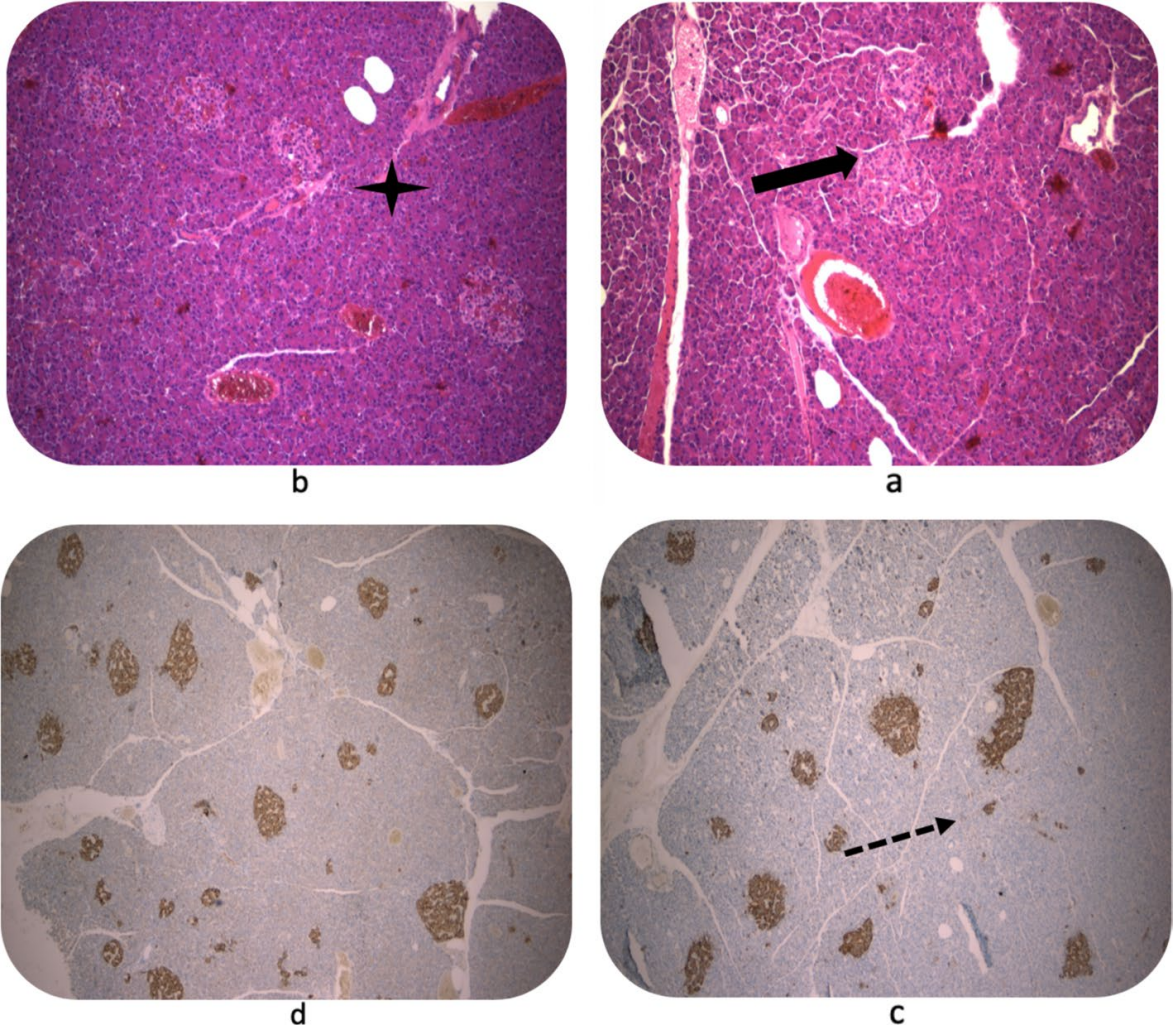
Pathological report of the surgical sample. **A** and **B** TECHNIQUE: HEMATOXILINA- EOSNA. 10x magnification Pancreatic parenchyma of globally preserved histoarchitecture is observed, with the presence of islets of Langerhans cells of different sizes, some with a tendency to cluster (arrow in fig. 2a), and in sectors with a certain tendency to adopt a periductal disposition (cross in fig. 2b). **C** and **D** IMMUNOHISTOCHEMICAL TECHNIQUE WITH CHROMOGRANINE. 4X INCREASE Positive staining (cytoplasmic and nuclear) is observed in pancreatic islet cells, which show different sizes and irregular shapes (fig c, arrow)

The patient had a satisfactory postoperative recovery and was discharged 3 days after the surgery took place. The surgical drain was removed on the 7th postoperative day after an imaging study showed absence of free abdominal fluid or intra-abdominal abscess. The final pathology report confirmed the presence of a brownish area with an irregular surface, within a homogeneous distal pancreas (size: 11.6 × 4.5 × 3 cm). No macroscopic nodular lesions were evidenced after successive serial cuts of the surgical specimen. Microscopically, numerous islets of Langerhans of variable sizes and irregular shapes were found: they were unevenly distributed, with a slight tendency to converge, and some of them had a periductal location (Fig. 2a, b, c, d). No tumor, or neoplastic cell proliferation were identified in the serial cuts from the studied material. Immunohistochemical techniques with chromogranin and synaptophysin were negative. Both morphological and immunohistochemical findings were compatible with diffuse hyperplasia of the islets of Langerhans, compatible with nesidioblastosis. Three months after surgery, patient remains with no surgical complications nor signs or symptoms consistent with hypoglycemia. Insulin, C-peptide levels and HbA1C percentage in blood were normal according to laboratory tests (Table 1).

### Systematic literature review

A systematic literature review was done by using as primary term “Nesidioblastosis”; 594 publications were shown. When the “adult” term was added, 277 publications could be obtained instead; when the term “adolescent” was used combined to the primary term, we obtained 63 publications. Adolescence has been defined as the transitional phase of growth and development between childhood and adulthood. The World Health Organization (WHO) defines an adolescent as any person between ages from 10 to 19. This age range falls within WHO’s definition of young people, which refers to individuals between ages from 10 to 24 [10].

**Fig. 3:**
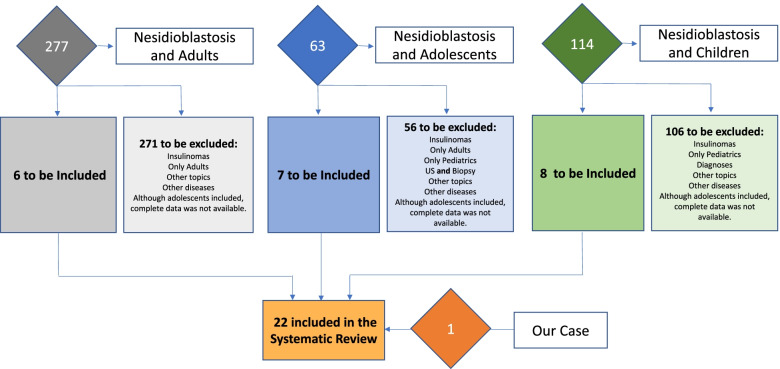
Systematic Review Flow-Chart

After this initial search, we decided to add the term “children”, considering that the broad spectrum of ages comprehended in the definition of adolescents might have them included both in pediatric or adult medical groups. The new search resulted in 114 additional manuscripts. Therefore, 454 final manuscripts were individually evaluated, and after matching them for duplications, a final number of 22 articles were included in the systematic review (Fig. 3). From 1980 to the present, from a total of 205 patients included in 22 manuscripts (9 case reports, 13 case series); 41 could be identified as adolescents (Table 2).

**Table 2:** Systematic review of the existing reports of nesidioblastosis in teenagers or adults

	Year	Nr of pts / Nr adoles.	Age	Sex	Medical treatment	Surgical treatment	Type of surgery	Resolved	Follow-up/survival
**Dahms B et al.** **[**11**]**	1980	16/1	15	M	-	+	SDP (2 procedures)	YES	5 years
**Harness JK et al.** **[**12**]**	1981	6/2	11 20	F M	+	+	SDP	YES	6 years 3 months
**Dunger DB et al.** **[**13**]**	1988	10/2	10 11	DNR	+	+	SDP	YES	NA
**Pedrazzoli S et al.** **[**14**]**	1994	58/1	16	M	-	+	DP	YES	4 years
**Rother KL et al.** **[**15**]**	1995	12/1	12	M	+	+	SDP	YES	NA
**Leibowitz G et al.** **[**16**]**	1995	14/11	11 to 24	8F	+ (11)	+ (8)	DP	YES	NA
**Fuller PJ et al.** **[**17**]**	1997	**1**	24	F	+	+	SDP	YES	2.5 years
**Dacou-Voutatakis C et al.** **[**18**]**	1998	15/5	10 to 20	DNR	+	+ (1)	SDP	YES	NA
**Ueda Y et al.** **[**19**]**	1998	**1**	23	M	-	+	DP	YES	2 years
**Van der Wall B et al.** **[**20**]**	1999	2/1	16	F	+	+	DP	YES	5 years
**Service FJ et al.** **[**21**]**	1999	5/1	16	M	-	+	SDP	YES	3 years
**Thompson G et al.** **[**22**]**	2000	10/1	16	M	-	+	SDP	YES	4 years
**Witeless RM et al.** **[**23**]**	2001	**1**	22	M	-	+	SDP	YES	21 years
**Martinez Ibannez V et al.** [24**]**	2002	29/2	10 12	DNR	+	1	SDP	YES	NA
**Bin-Abbas BS et al.** **[**25**]**	2004	**1**	14	F	+	-	-	Diabetes	8 years
**Anlauf M et al.** **[**26**]**	2005	15/3	18 20 21	F M M	+	+	SDP	2 YES	14 years
**Raffel A et al.** **[**27**]**	2007	4/1	18	M	+	+	SDP	YES	11.5 years
**Kovacs E et al.** **[**28**]**	2008	**1**	22	M	+	-	-	YES	NA
**Restrepo K et al.** **[**29**]**	2009	**1**	20	F	+	+	DP	YES	8 weeks ^a^
**Soares F et al.** **[**30**]**	2013	**1**	22	F	-	+	TP	YES	NA
**De Jesus et al.** **[**31**]**	2015	**1**	14	F	+	+	SDP	YES	2 years
**Castillo-Lopez** et al. **[**32**]**	2020	**1**	15	M	+	+	DP	YES	1 year
**Summary**	**1989 - 2020**	**205/41**	**10 to 24 years**	**-**	**34**	**32**	**23 SDP**	**39 yes**	**3 months to 21 years**

*TP *Total Pancreatectomy*, SDP *Subtotal - Distal Pancreatectomy*, DP *Distal Pancreatectomy*, DNR *Does not report*,  P *Pancreatectomy*, CDP *cephalic duodenopancreatectomy*.*

(^*^ 8 weeks after patient requires to have a TP to be completed, and the pathologist reports the existence of a 1.5 cm nodule positive for insulinoma with a 1% positive Ki-67).

Only 9 out of them responded to medical therapy and 32 required surgery. From the latter group, 72% received subtotal distal pancreatectomy (SDP) (60 to 80%) as primary treatment; which was done in 2 surgeries in 2 patients that did not resolve after the first one. Asymptomatic long-term follow-up has been reported in the vast majority of cases with follow-ups up to 21 years.

## Discussion

Hyperinsulinemic hypoglycemia is a challenging condition to treat. It is characterized by unregulated insulin release, leading to persistently low blood glucose concentrations, which increases the risk of neurological damage in these patients. In our case report, patient presented evident symptomatic hypoglycemia at night, which resolved after any form of glucose intake [33].

For the differential diagnosis of persistent HH, imaging studies can guide physicians to the focal or diffuse type of disease. Abdominal CT or MRI, remain the first-line option to visualize an insulinoma [34]; although other techniques like octreotide scintigraphy, glucagon-like peptide type 1 *s*cintigraphy (GLP-1) can be performed, they have low sensitivity [35, 36, 37]. If diffuse disease is part of the working diagnosis, imaging with the ^18^F-DOPA-PET-CT scan should be performed; and it has become the preferred study for most patients, including those with a medically unresponsive disease, who are thought to have a focal lesion, in order to identify its precise location before surgery [38]. Genetic mutation testing has become standard of care for prognosis, family counselling and as a diagnostic tool when a focal pancreatic lesion is present [39].

In our case, he underwent an 18-F-DOPA PET scan, which showed findings consistent with diffuse hyperfunctioning of the pancreatic tissue in body and tail.

Diazoxide is the mainstay of medical management, it acts by inhibiting glucose stimulated insulin secretion; and although it is used to try to control symptoms, in most cases relief cannot be observed, as mentioned in our case. Octreotide, a long-acting analog of somatostatin, inhibits insulin release and may control hypoglycemia in patients in whom diazoxide has failed [18].

It is important to rule out Munchausen syndrome or factitious disorder before surgical decision making. During the assessment of pediatric patients, psychological evaluation of the patient and its family should be part of the pre-surgical workup, in our setting it was not possible to correlate studies determining the use of sulfonylureas in urine and blood, nor the use of insulin.

In the real life we could not rule out Munchausen syndrome, and neither it there was evidence of elevated c- peptide; therefore, the surgical decision was made based on the patient’s clinical course and the lack of non-response to pharmacotherapy; in addition to the hyperfunction of the pancreas observed in the Pet scan, and assessing the increase life threating risk.

The post-surgical outcome validates the decision made, since hypoglycemic episodes resolved and the patient is currently metabolically stable, with a significant improvement in his quality of life.

In our systematic review, 34/41 patients received medical therapy, but only 9 (26,4%) were able to maintain long term control without requiring surgery, and 1 was reported as becoming diabetic. The other 32 received surgery, the one most commonly performed is SDP in 72% of the surgical patients. This procedure requires to remove between 60 to 80% of the pancreas, less than what has been recommended in newborns or children (up to 95%) [18]. In children early and aggressive surgery has been recommended to decrease neurological sequelae; but the longer follow up of these patients has shown that diabetes mellitus and linear growth impairment were frequent complications of this extensive surgery. In adolescents, the biggest surgical challenge is to perform a surgical resection able to guarantee long term symptomatic control without evolving into diabetes. This is one of the reasons why, although minor (1/23 pts), there is always a risk of requiring a second operation to extend the pancreatectomy if symptoms are not controlled with the first one; patients and parents should be notified about this alternative before surgery, and it should be part of the surgical consent [27] as well. Although the adolescent form of the disease resembles the adult form, and since there is no optimum procedure for all adolescent or adult patients with nesidioblastosis, patients should understand that a second operation involves less risk, compared to the risk of lifetime diabetes mellitus, after functional and extensive pancreatectomy [22].

Current and evolving PET-CT radioisotopes will help to maximize the chances for differential diagnoses with insulinoma. Although the response rate is low, medical therapy remains the initial therapy; but as soon as the lack of response is identified, surgery should be indicated, as the last but only therapy able to provide long term control of the disease in the vast majority of patients. Experienced surgical teams should minimize the risk of suffering from post-surgical diabetes [40–47].

Surgery was able to assure long term control of the non-insulinoma pancreatogenesis of the hypoglycemia syndrome in the vast majority of patients. As part of the findings of the systematic review, only one patient had simultaneous nesidioblastosis and insulinoma; found in the second surgery requiring total pancreatectomy [29]. Except for this particular case, no patient from the whole revision, including our case, was found to have a discrete insulinoma; however, the resected pancreas demonstrates the presence of islets hypertrophy.

A great limiting factor in the assessment of our patient was the impossibility of performing genetic tests before surgery; understanding that according to the literature they have become a new fundamental diagnostic pillar for the diagnosis and management of HH in children and adults.

The systematic review showed us that more adolescents are suffering from HH than what a simple search allows to identify. However, they have been included in reports from pediatric centers when their ages ranged from 10 to 16 years, while the older (16 to 24 years) were reported in manuscripts as adult patients because they were referred to adult surgical teams.

## Conclusion

Hyperinsulinemic hypoglycemia remains a clinical challenge to be diagnosed, as well as a difficult pathology to distinguish from insulinoma or another insulin-producing tumor. Both diseases develop symptomatic hypoglycemia and endogenous hyperinsulinism, and both might have lesions found in imaging studies.

Although symptoms generally resolve upon glucose intake, hypoglycemia can be life-threatening for all ages. In addition, it is well known that severe hypoglycemia increases cardiovascular risk and worsens cognitive impairment.

Besides the development of new diagnostic tools and therapeutic agents, when HH with nesidioblastosis is suspected, multidisciplinary management is recommended. Treatment should begin with strict low carbohydrate diet, followed by medication therapy. When other treatment options fail, surgical options can be considered as a subtotal pancreatectomy has proven to be a safe and effective long lasting treatment for patients with HH.

## Data Availability

The datasets used and/or analysed during the current study are available from the corresponding author on reasonable request.

## Abbreviations

HH: Hyperinsulinemic hypoglycemia
US: Ultrasound
CT: Computer Tomography
PET scan: Positron Emission Tomography scan
18-F-DOPA: Fluoro-18-L-dihydroxyphenylalanine
WHO: World Health Organization
TP: Total Pancreatectomy
SDP: Subtotal - Distal Pancreatectomy
DP: Distal Pancreatectomy
DNR: Does not report
CDP: Cephalic duodenopancreatectomy
GLP-1: Glucagon-like peptide type 1 scintigraphy
